# Use of a Novel Surgical Irrigant Significantly Reduces Rate of Infection in Primary Hip and Knee Arthroplasty at 1 year

**DOI:** 10.1016/j.artd.2025.101907

**Published:** 2025-11-26

**Authors:** Ravi K. Bashyal, Avinash Inabathula, Samantha Lariosa, S. David Stulberg

**Affiliations:** a Department of Orthopaedic Surgery, Endeavor Health Orthopaedic & Spine Institute, Skokie, IL, USA; b Department of Orthopaedic Surgery, Northwestern University Feinberg School of Medicine, Chicago, IL, USA

**Keywords:** Periprosthetic joint infection, Surgical site infection, Povidone–iodine, Citric acid, Xperience, Arthroplasty

## Abstract

**Background:**

Periprosthetic joint infection (PJI) prevention in primary hip and knee arthroplasty remains an important challenge in arthroplasty. Dilute topical povidone–iodine followed by a sterile saline rinse is widely used for intraoperative irrigation and infection prophylaxis in surgery. The Food and Drug Administration recently issued a reminder about the nonsterility of topical iodine preparations used in deep surgical wounds. Thus, our institution sought a terminally sterilized alternative. We investigated a terminally sterile irrigant with minimal cytotoxicity, efficacy against biofilm, and no required secondary rinse.

**Methods:**

This was a single-surgeon retrospective cohort study of 2087 consecutive primary total hip and knee arthroplasties with minimum 1-year follow-up in a major metropolitan community hospital. The control group of 1045 patients received a dilute povidone-iodine soak followed by saline rinse. The experimental group of 1042 patients received the new irrigant (XPerience (XP)) without secondary rinse. The International Consensus Meeting 2018 recommended algorithm and criteria were used to diagnose PJI.

**Results:**

Overall, the PJI rate was 0% (0 of 1042) in the XP group and 0.6% (6/1045) in the povidone-iodine group (*P* = .017). The overall return to operating room rate was 0.5% (5 of 1042) in the XP group and 1.1% (12 of 1045) in the control group (*P* = .11).

**Conclusions:**

The novel solution had a lower infection rate in our cohort. We conclude that it is a comparable alternative to povidone–iodine. An ongoing prospective randomized control trial and a cost-benefit analysis may provide stronger guidance for surgeons.

## Introduction

Infection prophylaxis is an active area of study given the devastating nature of periprosthetic joint infection (PJI). Despite many new prevention strategies, there has not been a consistent decrease in PJI rates over the last 15 years in the United States [1]. Further innovation and investigation are still warranted in this space. Intraoperative irrigation with antiseptic agents is common practice in total hip arthroplasty (THA) and total knee arthroplasty (TKA). Several adjuvant solutions have been studied in-vitro and in animal PJI models [[2], [3], [4], [5], [6]]. Theoretically, these agents help prevent PJI by reducing bacterial burden during total joint arthroplasty (TJA), although quality prospective studies in this area are lacking. Many irrigants exhibit varying degrees of cytotoxicity if left in contact with host tissues, often requiring secondary rinsing with saline after application [5,7,8]. This greatly limits the duration of activity and reduces concentrations well below their active level. In unicompartmental knee arthroplasty (UKA), some authors recommend avoiding chondrotoxic irrigants all together [5,9].

The few published comparative irrigation studies have mixed results but several professional organizations endorse dilute povidone-iodine (PVP-I), including the 2017 International Consensus Meeting (ICM) Clinical Practice Guidelines [7]. However, the Food and Drug Administration (FDA) recently issued a label change request to manufacturers and a reminder to health-care professionals stating, “If a product does not state “sterile” on the label, health-care professionals should be aware that they are using a nonsterile product” [10,11]. After previous studies demonstrated topical PVP-I irrigation followed by saline rinse reduced PJI rates compared to saline irrigation at minimal added cost, many institutions adopted it as their antiseptic of choice [[11], [12], [13], [14]]. Although they are bactericidal, manufacture of many “external use only” antiseptics does not include sterilization and postsurgical infection outbreaks have occurred due to contamination of topical solutions [10,15,16]. The FDA also recommended against dilution of topical PVP-I stating, “These products also should not be diluted after opening” [11]. Moreover, in-vitro studies have shown dose and time dependent toxicity of PVP-I to fibroblasts and osteoblasts at concentrations over 0.3%, though this has not been linked to adverse clinical outcomes [[17], [18], [19]]. The protocol for currently available irrigants also typically includes saline rinse after application, which reduces cytotoxicity but limits duration of action. No large clinical studies on irrigants, to date, have shown a statistically significant reduction in PJI compared to PVP-I in primary TJA. A noncytotoxic irrigation solution that does not require a secondary rinse may be beneficial in combatting PJI.

We previously showed that a citric acid–based solution, XPerience (XP) (Osartis GmbH, Münster, Hessen, Germany) had significant activity against planktonic and sessile bacteria and fungi in-vitro [6]. In this study, XP inhibited biofilm regrowth starting at 5 minutes and up to 5 hours [6]. It demonstrated potent activity against common biofilm-forming organisms including *Pseudomonas aeruginosa*, *Staphylococcus aureus*, and *Enterococcus* species [6]. Based on our prior study, other literature supporting efficacy of XP, and the FDA statements concerning dilute topical PVP-I, we decided to study XP further at our institution [6,11,20]. This terminally sterilized solution of citric acid (3.25%), sodium citrate (3.13%), and sodium lauryl sulfate (0.1%) in sterile water showed no in-vitro toxicity to human cells at the concentrations used, even with prolonged contact time [21]. Citric acid is approved for use in endodontic surgery as a bone surface cleanser and has shown no cytotoxicity or decalcification at the concentrations present in XP [[21], [22], [23], [24], [25]]. Citric acid’s antibacterial properties and induction of transforming growth factor-β1 release have also been demonstrated in-vitro [24,26]. It may also act as a chelator of metal ions in the extracellular polymeric substance of biofilms [27]. Sodium lauryl sulfate, is an antimicrobial surfactant that reduces biofilm surface tension and increases permeability [6]. We hypothesized that patients irrigated with XP would have a lower rate of PJI at 1 year post-TJA compared to dilute PVP-I irrigation.

It is important to note that many of the most commonly used commercially available irrigants in the United States, including, Xperience (Osartis – US distribution Osteoremedies), Irrisept (Irrimax corporation), Surgiphor (Becton Dickinson) and Prontosan (B. Braun) are FDA classified as “wound irrigation solutions or jet lavage solutions” under the product code “FRO”. This means they are regulated as unclassified medical devices requiring premarket notification, rather than classified drugs. The 510(k) approval for these products allows on label use for “intended mechanical cleansing and removal of debris, dirt, and foreign materials, including microorganisms, from wounds”. FDA regulation of antimicrobial irrigants is an ongoing area of discussion [28].

## Material and methods

This is a single-surgeon retrospective cohort study of 2087 consecutive patients undergoing primary elective THA or TKA at a large high-arthroplasty volume community hospital. Eligible patients were identified in our regional electronic database using Current Procedural Terminology codes. Minimum 1-year of follow-up was required for inclusion. Exclusion criteria included patients with prior native joint sepsis, UKAs (as dilute Betadine was avoided in these cases at our institution), and patients who did not receive iodine irrigation due to allergy. Patient age, body mass index (BMI), gender, American Society of Anesthesiologists (ASA) score, payor status (Medicare vs commercial vs public-aid), and anesthesia type were recorded. Comorbidities such as diabetes (type 1 or 2), cardiovascular disease (congestive heart failure, previous myocardial infarction, or peripheral vascular disease), ASA physical status score, and nicotine use (vaping, smoking, chewing tobacco, nicotine gum, etc.) were queried in the preoperative history and physical notes in our multi-institution electronic medical record system. Patients were counseled to cease nicotine products at least 4 weeks prior in order to reduce infection risk. However we did not routinely perform nicotine tests as a condition for surgery and patients [29].

In the control group, a total of 3-L sterile saline was used via pulse lavage throughout the case for visualization, bone preparation for cementing, and rinse-out of the PVP-I. A pulsatile lavage device and tip with maximum pressure of 15 pounds per square inch (low pressure) was used per the manufacturer guidelines [[30], [31], [32]]. These 1045 consecutive patients (492 THAs and 553 TKAs) from April 22, 2019, to November 30, 2021, were treated with a 500-mL dilute (0.35%) PVP-I soak for at least 3 minutes at the end of the case and prior to closure. The soak solution was prepared in a sterile basin of saline with measured aliquots of topical PVP-I. Following the soak, the remaining Betadine was rinsed with saline pulsed lavage prior to arthrotomy closure. From December 1, 2021, to January 31, 2024, 1042 consecutive patients (456 THAs and 586 TKAs) were irrigated with 1 L of XP solution. XP was suctioned as saline would be during visualization and bone preparation. Then, after final implant placement and prior to closure, the remainder of the XP was applied via the same pulsatile lavage and suctioned out of the wound without soaking time—essentially used in the same manner as sterile saline would be. In line with the intended use of this product, no secondary rinse was done and arthrotomy and wound were closed.

All other perioperative protocols were identical between groups. Both groups followed standardized institutional multimodal infection prophylaxis protocols at our hospital. Nasal methicillin-resistant *staphylococcus aureus* colonization screening and risk factor comanagement/optimization alongside their primary care physician was done. All patients underwent chlorhexidine (CHG)-based skin preparation and shaving at the surgical site with clippers on the day of surgery. Patients received a prophylactic weight-based dose of intravenous cefazolin within 1 hour prior to incision. If a patient had history of true cephalosporin allergy or methicillin-resistant *staphylococcus aureus* colonization, they received 1 g of intravenous (IV) vancomycin prior to incision. All TKAs in both groups were cemented. Total hip stems were cemented only in cases of very osteoporotic bone (<1% in both cohorts). Arthroplasty was performed through posterior hip and medial parapatellar knee approaches. Gentamicin loaded high-viscosity bone cement (Palacos R+G, Heraeus Medical, Hanau, Germany) was used for all TKAs and cemented THAs in both groups. No drains were used in either cohort. No further post-operative IV antibiotics were given in either cohort. Postsurgical anticoagulation was directed by the patient’s Caprini score for venous thromboembolism in both groups [33]. Patients who scored ≥ 10 (high risk for venous thromboembolism) were placed on warfarin starting day 0 with low-molecular-weight heparin bridge until they achieved target international normalized ratio was 1.8-2.2. Patients with a score <10 received aspirin 81 mg twice daily alone [33]. There were no significant differences in anticoagulation protocols between groups. All patients received an IV dose of 1 g tranexamic acid prior to incision.

Primary outcomes of interest were all-cause reoperations (identified from our hospital electronic medical record as well as a review of outside hospital electronic medical records captured in our regional multinetwork electronic system) and PJIs at 1 year. All reoperations were first identified and these charts were carefully reviewed for the indication(s). In our practice, diagnosis of PJI was based the validated algorithm and lab cutoffs recommended by the 2018 ICM on Orthopedic Infections [34,35]. The decision to aspirate a patient presenting with joint pain was based on the recommended factors: history and physical exam: clinical findings (increasing pain, fevers, swelling, erythema, warmth, and/or persistent drainage), elevated serum C-reactive protein (CRP) (>10.0 mg/L), or serum erythrocyte sedimentation rate (>30 mm/hour) [34,36,37]. The guidelines established major criteria (2 positive cultures of the same organism or sinus tract communicating with the joint) and minor criteria with weighted scores. The meeting recommended differing cutoffs for serum and synovial fluid labs based on time since surgery (<90 days considered acute and >90 days considered chronic). Within 90 days of surgery, minor criteria cutoffs were as follows: elevated serum CRP (>100 mg/L), synovial white blood cell count (>10,000 cells/uL), and synovial polymorphonuclear cell % >90% [38,39]. After 90 days from surgery, minor criteria cutoffs were as follows: elevated serum erythrocyte sedimentation rate (>100 mm/h), elevated serum CRP (>10 mg/L), synovial white blood cell count (>3000 cells/uL), and synovial polymorphonuclear cell % >80. Those meeting any of the major criteria or those with a minor criteria score totaling ≥ 6 are treated for infection. Patients with no major criteria and inconclusive scores (2-5) were still taken to surgery and intraoperative fluid and tissue pathology was used to help confirm diagnosis [35]. All patients with diagnosed PJI were evaluated by infectious disease and received IV antibiotic treatment based on culture and sensitivity results. Hematomas which required irrigation and debridement were considered reoperations. None of these patients received modular component exchange. All hematomas that went to the operating room were sent for cultures, polymerase chain reaction testing, and cell counts to ensure there were no superimposed infections. Patients undergoing reoperation for aseptic causes received no additional antibiotics beyond 24 hours after surgery.

Reoperations and complications were identified during retrospective review by querying our database for relevant International Classification of Diseases-10 codes and careful chart review of all patients. We also queried a regional multinetwork electronic system to see if patients may have been treated for complications at outside hospitals. Continuous variables were tested using a t-test and categorical variables were tested using Pearson’s Chi-Squared tests or Fisher’s Exact test when necessary. Fisher’s Exact test was used to compare incidence of infection due to rarity of the event.

## Results

Both cohorts were comparable in terms of BMI, ASA score, and diabetes (Table 1). Most patients were ASA 2 or 3 with average BMI of 30.0 kg/m^2^ in the XP group and 29.8 in the PVP-I group. Distribution of THAs and TKAs was not different between groups (53% TKAs +47% THAs vs 56% TKAs +44% THAs, *P* = .13). Operative times were also similar between groups (mean 67 minutes vs 65 minutes, *P* = .54).

**Table 1:** Patient characteristics between groups.

Variable	Control, N = 1045^a^	XP, N = 1,042^a^	*P* value^b^
Age	68 (10)	68 (9)	.12
Gender			.7
Female	636 (61%)	616 (59%)	
Male	409 (39%)	426 (41%)	
BMI (kg/m^2^)	29.8 (5.4)	30.0 (5.6)	.3
ASA			.7
1	29 (2.8%)	39 (3.7%)	
2	660 (63.2%)	608 (58%)	
3	351 (33.6%)	385 (37%)	
4	4 (0.4%)	10 (1.0%)	
Nicotine use (within 6 wks)	32 (3.1%)	57 (5.5%)	**.016**
Cardiovascular disease	240 (23%)	164 (28%)	.4
Diabetes	204 (20%)	192 (18%)	.2

Bold values indicate statistical significance.

a Mean (SD); n (%).

b Welch 2-Sample t-test; Pearson's Chi-squared test; Fisher's exact test.

Patients irrigated with XP solution saw a statistically significant reduction in PJI compared to the control group at 1 year (0% vs 0.6%, *P* = .017) (Table 2). There were 6 PJIs in the control group which all underwent surgical intervention and 0 in the XP group (Table 3) at minimum 1-year follow-up. Most (5 of 6) infections occurred within the first 90 days after surgery and all were deep infections. All reoperations for infection in this cohort had CRP >40 mg/L (well above the recommended cutoff) and organisms were isolated on intraoperative cultures. There was no statistically significant difference in number of hematomas requiring reoperation between groups (Table 3, *P* = .32). All hematoma preoperative and intraoperative cultures were negative and they did not meet the ICM 2018 criteria [34]. None of the hematoma patients developed subsequent signs of PJI after irrigation and debridement.

**Table 2:** Infections and reoperations at 1-y follow-up.

Variable	Control, N = 1045^a^	XP, N = 1,042^a^	*P* value^b^
PJIs	6 (0.6%)	0 (0%)	**.017**
Reoperations	12 (1.1%)	5 (0.5%)	.11

Bold values indicate statistical significance.

a n (%).

b Fisher's exact test; Pearson's Chi-squared test.

**Table 3:** Summary of reoperations at 1 y.

Joint	Procedure	Time from index surgery	Cultured organism (if applicable)
Dilute PVP-I, N = 1045			
TKA	Closed reduction and revision after trauma	3 d	n/a
TKA	Hematoma I&D	6 d	Negative cultures and PCR
TKA	Hematoma I&D	21 d	Negative cultures and PCR
THA	Hematoma I&D	1 mo	Negative cultures and PCR
THA	Revision B2 fracture	5 d	n/a
THA	Revision B2 fracture	1 mo	n/a
TKA	Acute infection: I&D poly exchange	10 d	Serratia marcescens
TKA	Acute infection: I&D poly exchange	24 d	Enterococcus faecalis
TKA	Acute infection: I&D poly exchange	1 mo	E. coli
TKA	Acute infection: I&D poly exchange	1 mo	MSSA
THA	Acute infection: I&D poly exchange	5 wks	Enterobacter cloacae
THA	Chronic infection: 2-stage revision	4 mo	P. Acnes
XP, N = 1042			
TKA	Hematoma I&D	3 wks	Negative cultures and PCR
THA	ORIF Greater troch fracture	1 mo	n/a
THA	Revision for B2 fracture	1 mo	n/a
TKA	Repair of traumatic wound dehiscence	12 d	n/a – superficial closure only
THA	Head and liner exchange for instability	2 mo	n/a

I&D, irrigation and debridement; MSSA, methicillin susceptible *staphylococcus aureus*; PCR, polymerase chain reaction*.*

All-cause readmission rates were also documented. There were 33 of 1045 (3.2%) readmissions in the control group and 31 of 1042 (3.0%) in the XP group (*P* = .8). The causes of readmission were predominantly for postoperative medical complications such as deep vein thrombosis, bleeding, urinary retention, and pneumonia. We did not find any adverse reactions attributable to either irrigation solution.

## Discussion

Study results were consistent with our hypothesis that XP would reduce incidence of PJI. This is the largest retrospective clinical comparison study of a novel irrigant in primary TJA against PVP-I. To date, XP is the only solution to show statistically significant acute PJI reduction compared to PVP-I. Taking our study’s infection proportions, 1 infection is prevented for every 167 TJAs performed with XP instead of PVP-I (number needed to treat). This reduction in acute PJI may be due to 2 factors: the effectiveness XP against planktonic and sessile bacteria and its lack of cytotoxicity, allowing persistent antimicrobial activity for up to 5 hours after application [5,8,40]. We contend our cohort is representative of a diverse major metropolitan arthroplasty population (Table 1). Payor status is another key variable affecting outcomes in patient populations as Medicare and Medicaid patients have been shown to be at increased risk for PJI [41,42]. Distributions of Medicare, Medicaid, and commercially insured patients were similar between study groups. Dilute iodine is an accepted adjuvant irrigant in arthroplasty nationwide. Our observed infection rate with dilute povidone iodine was low and consistent with recent large studies on primary TJA [40,43]. In light of recent FDA statements on nonsterile topical antiseptics and FDA recommendation against dilution of these products, arthroplasty surgeons are reconsidering topical PVP-I. A sterilized 0.5% povidone iodine solution is now available but still requires rinsing after application, limiting duration of action [44]. Authors have studied various concentrations of PVP-I, but little clinical data exist to support use of a particular concentration. A 0.35% dilution soak for 3 minutes was used in this study, which is comparable to protocols in most studies [5]. Very rarely (∼0.4%), patients may develop an allergy to copolymers present in povidone, the synthetic carrier molecule in PVP-I [45]. Although the relevance of this at diluted concentrations is unknown, some authors recommend against use of povidone irrigation in such patients [3]. On the other hand, true allergy to constituents of XP is very rare as well and there have been no reported allergic reactions to date. Lauryl sulfate can cause skin irritation in patients who have been hypersensitized from chronic repeated exposure. However, true allergy has not been reported and it does not cross-react in sulfonamide allergic patients [46].

CHG is another increasingly used irrigant, though the optimal concentration for bactericidal vs cytotoxic activity is unknown. It has shown excellent in-vitro efficacy but is still clinically under evaluation. A sterile FDA-cleared CHG formulation is available for intrawound irrigation. While some studies support efficacy of CHG, a recent large retrospective study found no difference in infection rate compared to dilute PVP-I (0.338%) [47,48]. An in-vitro study from van Meurs, et al raised concerns regarding chondrotoxicity and osteoblastic precursor toxicity at the concentrations required for bactericidal activity with both PVP-I and CHG (after 2 minutes of exposure) [49]. CHG was cytotoxic even at low concentrations, leading some surgeons to avoid it in UKA out of concern for chondrotoxicity. Given the favorable in-vitro safety profile of citric acid even at 10% concentration, XP is used for UKAs at our institution [5]. Benzalkonium chloride solution with acetic acid is also seeing use in arthroplasty, as it lowers local pH and effectively inhibits bacterial function on metal surfaces [50]. However, large comparative studies on this agent in primary TJA are lacking. In-vitro studies demonstrate a minimum 20 minutes of exposure at 5% concentration was required for biofilm eradication, with higher concentrations damaging tissues [51]. Another in-vitro study on human fibroblasts showed significant reduction in viability after only 1 minute of exposure to CHG or benzalkonium solutions [8]. A secondary saline rinse is recommended by manufacturers for both CHG and benzalkonium chloride to limit their cytotoxic effects.

In addition to toxicity, cost to patients is also worth considering in today’s increasingly cost driven market. The burden of PJI in America is expected to rise to $1.85 billion by 2030 [52]. The lifetime cost of a single PJI was estimated at over $300,000 per patient with a direct cost at $100,000 [53]. A future cost-benefit analysis of XP based on our number needed to treat for PJI would be helpful in determining its cost-effectiveness relative to the known cost-effectiveness of PVP-I [12].

This study provides a meaningful addition to existing data on irrigants in TJA. A recent retrospective study found improved range of motion, postoperative swelling, and lower assistive device use at 2 weeks with XP compared to PVP-I [20]. This may be consistent with in-vitro studies showing citric acid promotes release of transforming growth factor-β1, a mesenchymal stem cell attractant and important immunomodulator [23,54]. Citrate is also a chelator of metal ions, including free calcium ions that play key roles in cytokine release and physiologic homeostasis. Local and systemic anti-inflammatory effects of calcium chelation by citrate in XP warrant further study.

This study’s key limitations are that it is a nonrandomized, retrospective, single institution cohort with noncontiguous controls. However, a single-surgeon study also controls for variability in technique and perioperative protocols. It is worth noting that odds ratio for PJI cannot be calculated from our data since the event rate in the XP group is zero. Our obtained result carries a fragility index of 1 [55]. This limits our ability to draw strong conclusions regarding superiority, as is the case for many studies with low infection rates. Longer follow-up may better define odds ratio of infection between groups, though we do not believe intraoperative irrigant choice would meaningfully affect the risk of late infection.

With regards to diagnosis of infection, the International Consensus on Orthopedic Infections guidelines provided a sensitive and specific algorithm with thresholds for diagnosing the vast majority of infections [36,[56], [57], [58]]. Despite using a peer-reviewed systematic approach with high sensitivity and specificity for diagnosing infection, it is always possible, that occult infections were treated as aseptic revision [36,37]. It would be unlikely for such infections to resolve on their own without antibiotic treatment, however. None of the patients who underwent reoperation for aseptic causes (including hematomas) developed subsequent PJIs to date and none were kept on suppressive antibiotics. Hematoma patients all had returned for 1-year follow-ups with good outcomes and no subsequent pain or infection. Loss to follow-up is another weakness inherent in any longitudinal study but this was minimal in our study. We also queried our multi-institutional regional database to check for patients who may have been treated at other institutions.

## Conclusions

This retrospective study demonstrates a lower rate of PJI with XP compared to dilute PVP-I at our institution. To date, there have been no similarly large comparative studies on irrigation solutions in primary TJA with 1-year follow-up. We contend that XP is an acceptable alternative to PVP-I given its biofilm destroying ability, extended duration of action, excellent safety profile, and seamless integration into any workflow. Moreover, it does not pose a significant added cost compared to alternatives and may even confer savings from prevented PJI. A prospective randomized controlled trial testing XP against PVP-I with PJI as the primary endpoint is ongoing and may provide further guidance for surgeons after completion.

## Conflicts of interest

Ravi K. Bashyal is on the speakers' bureau/receives paid presentations for and is a paid consultant for Smith & Nephew, Stryker, and NextScience.

S. David Stulberg receives royalties from Innomed and Stryker and owns stock or stock options and is an unpaid consultant at TracPatch LLC.

The other authors declare no potential conflicts of interest.

For full disclosure statements refer to https://doi.org/10.1016/j.artd.2025.101907.

## CRediT authorship contribution statement

**Ravi K. Bashyal:** Writing – review & editing, Writing – original draft, Supervision, Project administration, Methodology, Investigation, Data curation, Conceptualization. **Avinash Inabathula:** Writing – review & editing, Writing – original draft, Project administration, Methodology, Investigation, Formal analysis, Conceptualization. **Samantha Lariosa:** Project administration, Formal analysis, Data curation. **S. David Stulberg:** Writing – review & editing, Supervision, Methodology, Investigation, Conceptualization.

## Appendix A Supplementary data

Conflict of Interest Statement for Lariosa

Conflict of Interest Statement for Bashyal

Conflict of Interest Statement for Inabathula

Conflict of Interest Statement for Stulberg
