# Supplementary material for: Use of a Novel Surgical Irrigant Significantly Reduces Rate of Infection in Primary Hip and Knee Arthroplasty at 1 year
Source: Arthroplast Today. 2025 Nov 26;36:101907. doi: 10.1016/j.artd.2025.101907 (PMC12702035; doi:10.1016/j.artd.2025.101907)
Supplement: Conflict of Interest Statement for Bashyal [file mmc2.pdf]

INDIVIDUAL CONFLICT OF INTEREST STATEMENT

American Association of Hip and Knee Surgeons

(Adopted from the American Academy of Orthopaedic Surgeons disclosure statement)

The following form **must be filled out completely and submitted by each author (example, 6 authors, 6 forms). All items require a response. If there is no relevant disclosure for a given item, enter "*None*."**

Manuscript Title
Use of a Novel Surgical Irrigant Significantly Reduces Rate of Infection In Primary Hip and Knee Arthroplasty

1. Royalties from a company or supplier (The following conflicts were disclosed):

None

2. Speakers bureau/paid presentations for a company or supplier (The following conflicts were disclosed):

Smith-Nephew, Stryker, NextScience

3A. Paid employee for a company or supplier (The following conflicts were disclosed) :

None

3B. Paid consultant for a company or supplier (The following conflicts were disclosed):

Smith-Nephew, Stryker, NextScience

3C. Unpaid consultants for a company or supplier (The following conflicts were disclosed):

None

4. Stock or stock options in a company or supplier (The following conflicts were disclosed):

None

5. Research support from a company or supplier as a Principal Investigator (The following conflicts were disclosed):

None

6. Other financial or material support from a company or supplier (The following conflicts were disclosed):

None

7. Royalties, financial or material support from publishers (The following conflicts were disclosed):

None

8. Medical/Orthopaedic publications editorial/governing board (The following conflicts were disclosed):

None

9. Board member/committee appointments for a society (The following conflicts were disclosed):

None

Each author must sign AND print or type his/her name, date and submit a separate form

In addition, one BLINDED Conflict of Interest form (no author names used) should be submitted per manuscript with all author disclosures.

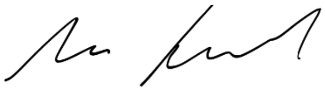

Ravi Bashyal		11/10/2024
Author Name (Print or Type)	Author Signature	Date
